# The resistome bridge between livestock and workers: novel frameworks for early detection and monitoring of antimicrobial resistance

**DOI:** 10.3389/fpubh.2026.1746385

**Published:** 2026-01-29

**Authors:** Silvia Vivarelli, Claudia De Francesco, Emilia Paba, Federica Giambò, Concettina Fenga

**Affiliations:** 1 Section of Occupational Medicine, Department of Biomedical and Dental Sciences, Morphological and Functional Imaging, University of Messina, Messina, Italy; 2 Department of Occupational and Environmental Medicine, Epidemiology and Hygiene, Italian Workers’ Compensation Authority (INAIL), Rome, Italy

**Keywords:** antimicrobial resistance, gut microbiome, livestock farming, one health, resistome

## Abstract

Antimicrobial resistance (AMR) poses a critical threat to global health, driven by the extensive use of antibiotics in both human medicine and livestock production. In the context of the One Health framework, this review investigates the role of the gut microbiome and resistome, which represents the collection of antimicrobial resistance genes (ARGs), within livestock and among occupationally exposed workers. Intensive farming practices often involve routine, subtherapeutic antibiotic use, fostering antibiotic-resistant bacteria (ARB) in the gastrointestinal tract of animals. These ARB and ARGs are excreted into the environment, contributing to resistance spread through mobile genetic elements. From a Planetary Health perspective, this environmental dissemination reflects how human-driven livestock practices can perturb ecosystems, creating global health risks that link animal, human, and environmental well-being. Human exposure, particularly among farm workers and veterinarians, raises significant concerns about zoonotic transmission of pathogens and, potentially, ARB. Novel advances in metagenomic and metatranscriptomic technologies enhanced our understanding of gut microbial communities and their resistomes, revealing overlaps in ARG profiles between animals and livestock workers. These technologies also support the development of novel microbiome-targeted strategies, including prebiotics, probiotics, food supplementation and workplace-improvement strategies, aimed at reducing antimicrobial use and restoring healthy microbiome balance. The review also highlights the importance of integrated surveillance and cross-sectoral collaboration to monitor and control AMR transmission. Understanding the ecological dynamics of the gut resistome in livestock systems is essential for designing effective interventions that safeguard both animal and human health.

## 1 Introduction

Antimicrobial resistance (AMR) represents a crucial global health threat and a pressing One Health challenge, with profound implications for humans, animals, and environment (1). The widespread and often indiscriminate use of antibiotics, both in human medicine and livestock production, has accelerated the emergence of antibiotic-resistant bacteria (ARB) and resistance genes (ARGs), undermining the efficacy of essential antimicrobial therapies (2).

In particular, livestock farming represents a major driver of ARB spreading due to the routine use of antibiotics not only for animal treatment, but also for prophylaxis and growth promotion. In high-intensity animal farming systems, antibiotics are frequently administered via feed or water, even in the absence of clinical disease (3). A significant proportion of these compounds is excreted unmetabolized, introducing both active residues and resistant microorganisms into the environment through manure. This creates potential reservoirs of ARGs in both soil and water systems, facilitating their spread via horizontal gene transfer through mobile genetic elements (MGEs) such as plasmids, transposons, and integrons. As a result, the gastrointestinal tract of livestock has become a well-recognized reservoir for ARB, shaped by both antibiotic exposure and environmental pressures (4).

Within livestock systems, the consequences of AMR spreading are not confined to animal health or productivity. Human populations, particularly those occupationally exposed such as farm workers or veterinarians, are at elevated risk of encountering ARB and ARGs through direct contact with animals, fecal matter, contaminated surfaces, and airborne particles. In turn, these exposed subjects can become a source of risk for other workers, as well as the general population (5).

These interactions raise concerns not only about environmental dissemination of resistance, but also about zoonotic transmission, based on the direct transfer of antibiotic-resistant pathogens from animals to humans. Pathogens such as *Escherichia coli*, *Salmonella enterica*, *Campylobacter jejuni*, and *Staphylococcus aureus* have been shown to carry transferable ARGs and they are capable of crossing the animal-human barrier, often leading to infections in humans that are increasingly difficult to treat. The dual role of these organisms as both zoonotic agents and ARG-vectors highlights the compounded threat they pose to public health (6).

This interconnectedness of AMR transmission across species and environments is central to the One Health framework, which emphasizes the inextricable links between human, animal, and environmental well-being. Within this paradigm, AMR is understood not as a series of isolated events, but as a system-wide phenomenon shaped by microbial ecology, antimicrobial usage patterns, and interspecies interactions. From a Planetary Health perspective, the spread of AMR in livestock systems exemplifies how human-driven practices can disrupt ecosystem integrity, contaminating soil and water microbiomes, hence contributing to a global health threat that transcends local environments (7).

Recent advances in high-throughput sequencing and metagenomics have provided powerful tools for investigating the microbial communities and AMR profiles within all different kind of ecosystems. In this context, the gut microbiome, a complex and dynamic microbial consortium, not only supports host’s nutrient metabolism and immune function but also acts as source of ARGs. Metagenomics and metatranscriptomics allow for the comprehensive characterization of the whole gut resistome, which represents the full complement of ARGs within gastro-intestinal microbial community, without reliance on culture-based methods (8). Importantly, the presence of ARB within the intestine can shape the composition and diversity of the gut microbiome, sometimes leading to dysbiosis in humans (9). Therefore, it is crucial to investigate studies addressing these interactions.

What is currently known about the relationship between gut microbial communities and ARGs in these settings? What insights do metagenomic studies provide into the composition of the intestinal resistome, and to what extent are resistome signatures shared between animals and humans? Finally, how can integrated surveillance and microbiome-based approaches within the One Health framework help contain the spread of AMR and mitigate the risk of zoonotic transmission? This review aims to explore these key questions within livestock farming and among occupationally exposed workers.

## 2 Methods

A keyword search was carried out in PubMed, Web of Science and Scopus databases. Keywords have been grouped in four different topics (i.e., Husbandry; Animals; Antimicrobial resistance; Microbiome). To perform the search, keywords within each topic were combined using the “OR” or “AND” operator. Search terms are summarized in Table 1. Furthermore, a MEDLINE search has been conducted using the Medical Subject Headings (MeSH), reported in Table 2. Papers published between 2014 and 2025 were considered.

**Table 1:** Summary of keywords used in the search.

Husbandry	Animals	Antimicrobial resistance	Microbiome
Livestock	Pigs	Antimicrobial	Microbiome
Farming	Porcine	Antimicrobial resistance	Gut microbiome
Farm	Swine	AMR (antimicrobial resistance)	Intestinal microbiome
Agriculture	Cows	Antibiotic resistance	Rumen microbiome
Barn	Cattle	Drug resistance	Gut flora
Stalls	Bovine	Resistant bacteria	Microbial community
Cage	Calves	Resistant genes	Microbiota
Backyard	Goats	Multidrug resistance	Intestinal flora
Grazing	Sheep	Antibiotic-resistant bacteria	Metagenome
Intensive farming	Ovine	ARGs (antibiotic resistance genes)	Metagenomics
Animal husbandry	Chickens	Mobile genetic elements	Metatranscriptome
Feedlot	Poultry	Horizontal gene transfer	Metatranscriptomics
Dairy farm	Broilers	Resistance transmission	Resistome
Slaughterhouse	Layers	Beta-lactam resistance	Gut resistome
Housing system	Hens	Colistin resistance	Microbial diversity
Pasture	Avian species	Vancomycin resistance	Microbial composition
Enclosure	Livestock species	Extended-spectrum beta-lactamases (ESBLs)	Taxonomic profiling
Manure management	Farm animals		Functional profiling
Stocking density			Shotgun sequencing
Animal production			16S rRNA sequencing

**Table 2:** Summary of medical subject headings (MeSH) terms used in the search.

Keywords	Husbandry/Animals	Antimicrobial resistance	Microbiome
MeSH heading	Animal husbandry (D000822)	Drug resistance, bacterial (D024881)	Microbiota (D064307), gastrointestinal microbiome (D000069196), metagenome (D054892)
Entry terms/scope	Includes breeding, feeding, care, housing animals	Includes beta-lactam resistance, ESBLs, multi-drug bacterial resistance	Includes microbial community, community structure, gut/intestinal flora
Subheadings/qualifiers	Classification; methods; standards; statistics	Genetics; drug effects; immunology; physiology	Drug effects; genetics; immunology; physiology
Hierarchical tree	J01.040.090	G06.099.225; G06.225.347	G06.591; G06.591.375; G05.360.340.550

**Figure 1:**
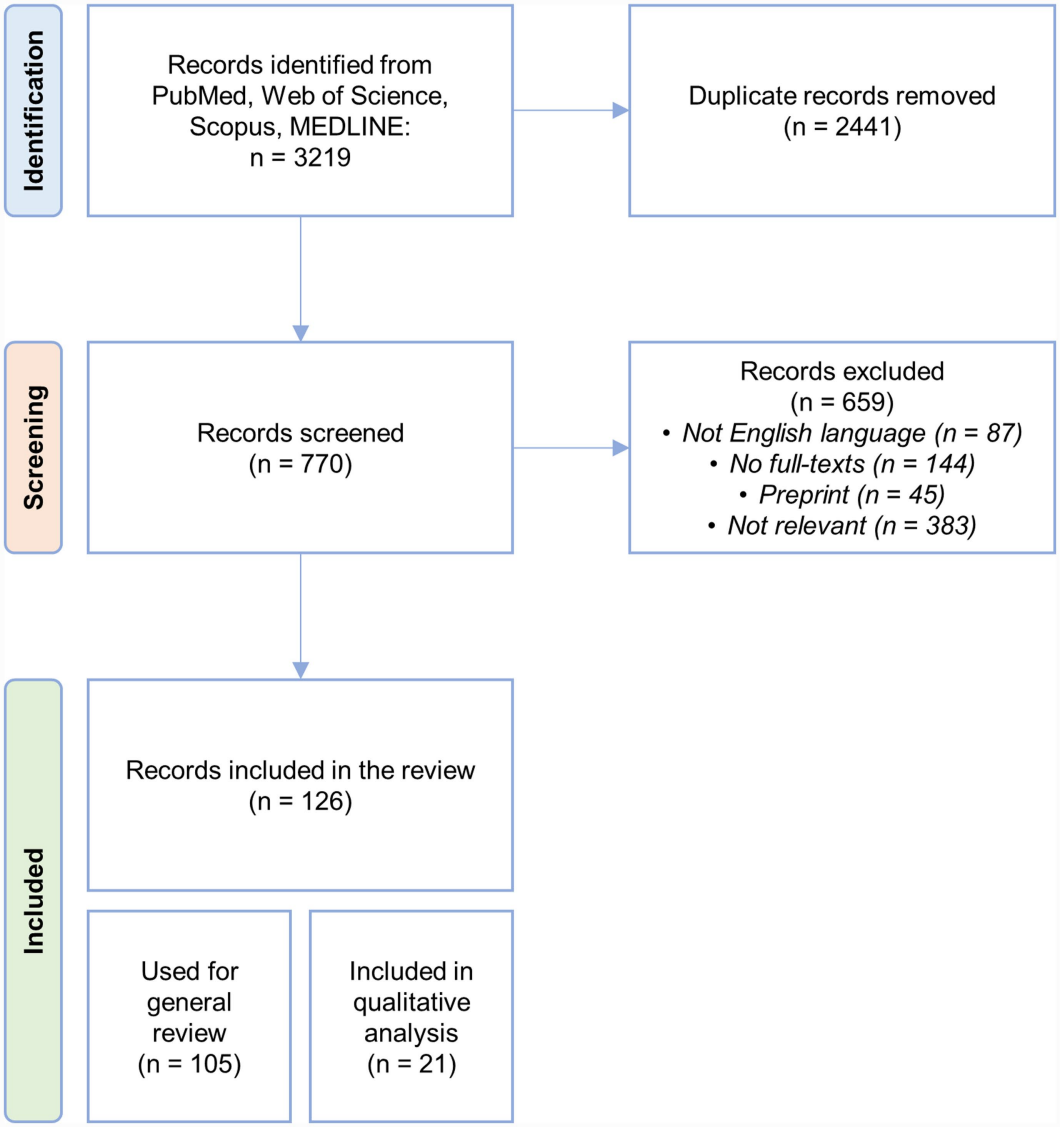
Study selection flow diagram. The diagram illustrates the process of study selection, including the number of records identified, screened, and finally included in the review. A total of 21 studies were included in the quantitative analysis.

As illustrated in the flow chart in Figure 1, the initial database search yielded a total of 3,219 records. After the removal of 2,441 duplicates, 770 records were screened. Of these, 659 articles were excluded for the following reasons: they were not published in English, full texts were unavailable, they were preprints, or they did not meet the predefined criteria in terms of relevance to the selected topics (i.e., Husbandry; Animals; Antimicrobial Resistance; Microbiome). Ultimately, 126 records were included in the final review. Among these, 21 were used in the qualitative analysis and specifically concern research studies presented in sessions 3.4 and 3.5, which focus, respectively, on gut microbiome and resistome analyses in livestock, and the impact of occupational exposure on the human gut microbiome and resistome.

## 3 Results and discussion

### 3.1 Antimicrobials use in livestock industry and health consequences

Antimicrobial drugs have long been considered as the cornerstone of modern medicine, revolutionizing the treatment of bacterial infections and drastically reducing mortality, particularly in vulnerable populations (10). Their role extends beyond curing infections to enable critical medical procedures. Within our era of advanced medicine, where robotic surgeries and immunosuppressive therapies are commonplace, antibiotics continue to safeguard patients from potentially lethal post-procedural infections. However, this very reliance has contributed to one of the severest public health crises of our time, the AMR (11).

ARB are able to evolve mechanisms to survive exposure to antibiotics that would normally inhibit or kill them. Multidrug resistance (MDR), where bacteria resist multiple classes of antibiotics, is increasingly common and is primarily driven by the overuse and misuse of these active molecules (12). This includes improper dosing, incomplete courses of treatment, and the use of antibiotics without prescription, which are practices especially prevalent in countries with weak regulatory oversight. The situation is exacerbated by the widespread and often indiscriminate use of antibiotics not only for human applications, but also in veterinary medicine, agriculture, and livestock production. In many countries, antibiotics are routinely added to animal feed to promote growth and prevent disease, even in the absence of clinical symptoms (13).

Up to 75% of these veterinary antibiotics is not metabolized by animals and is instead excreted into the environment through urine and feces. This waste, often used as fertilizer, introduces antibiotic residues and ARB into soil and water systems, thereby contaminating food crops and groundwater (14). This environmental dissemination facilitates the transfer of ARGs between bacteria, including those pathogenic to humans and associated with zoonoses, through various routes such as food handling, consumption of raw or undercooked animal products, as well as through direct contact with farm environments. Such gene transfer, mediated by MGEs like plasmids, transposons, and integrons, plays a critical role in the rapid and widespread emergence of resistant bacterial strains (15).

This issue first gained attention in the United Kingdom in the 1960s when the Swann Commission reported a connection between antibiotic use as growth promoters and the emergence of multidrug-resistant bacteria (16). As a result, the Commission recommended banning antibiotics relevant to human medicine for use in food-producing animals. Building on this, the European Union took significant action in 1999 by banning four antibiotic classes for growth promotion, and later expanded the ban to all antibiotic growth promoters by 2006 (17).

These legislative changes helped to moderate the issue. However, the transition away from antimicrobial drugs misuse has not been without challenges. Early studies from countries like Sweden and Denmark, which were early adopters of bans on antibiotic growth promoters, noted initial increases in animal health issues, especially for the gastro-intestinal system, such as diarrhea or mucositis in animals (18). Some producers also turned to high-dose zinc oxide as a dietary alternative to support gut health during the transition; however, this practice has now been banned in the EU due to environmental and antimicrobial-resistance concerns (19). These problems were ultimately addressed through improved livestock housing, hygiene, and overall farm management, indicating that non-antibiotic interventions can possibly mitigate the negative effects of antibiotic withdrawal (20).

In recent years, the European Union has reinforced its commitment to tackling AMR through comprehensive legislative and policy frameworks that emphasize a One Health approach. The 2017 EU Action Plan on AMR and its 2022 update within the Farm to Fork strategy have promoted integrated surveillance, responsible antimicrobial use in agriculture, and enhanced research into ARGs and the resistome (21). Regulation (EU) 2019/6 on veterinary medicinal products further restricts prophylactic antibiotic use in animals, supporting microbiome-friendly practices (22).

In this context, Italy has aligned its national strategies with EU directives, increasingly recognizing the importance of occupational and environmental exposure pathways (23). The Italian National Institute for Insurance against Accidents at Work (INAIL) plays a crucial role in this area by funding and coordinating research focused on the occupational risks associated with AMR, including studies of microbiota and resistome alterations in workers exposed to livestock environments (24–27).

Housing conditions and animal management have a significant influence on the spread of antibiotic resistance. Studies have shown that pigs raised in different types of housing had varying levels of resistant bacteria, suggesting that production environment plays a role in resistance patterns. Similarly, animals in denser living conditions exhibited higher resistance levels, reinforcing the importance of good management practices in reducing the need for antimicrobial treatments (28, 29).

AMR in livestock extends beyond animal health and productivity, posing significant social and economic challenges that affect human health as well as the environment. While AMR threatens the efficacy of curing infections in animals, its consequences ripple outward, influencing the broader public health landscape and ecological balance (16). Assessing the full scope of antimicrobial use and the burden of AMR in livestock is particularly complex due to the lack of standardized methodologies and the diverse nature of the data required. Despite these challenges, quantifying the impact of AMR is crucial. In the framework of the One Health approach, understanding and addressing both social and economic burden of AMR becomes an essential priority for sustainable animal breeding and global health security (30).

### 3.2 Molecular mechanisms driving AMR gene propagation

The antimicrobial resistance genes (ARGs) themselves can be highly specialized or broad in function. For instance, chromosomal mutations may target specific antibiotics (such as fluoroquinolones) by altering drug-binding sites on bacterial enzymes. Other mutations lead to the overproduction of efflux pumps, which expel a variety of antibiotics from bacterial cells, thus creating resistance to multiple drug classes simultaneously (31). These mechanisms are further complicated by the presence of MGEs, which enable bacteria to acquire resistance from unrelated species in a matter of hours, making AMR a global and cross-sectoral issue (32).

These resistance mechanisms can spread either vertically, through bacterial replication, or horizontally, through ARG transfer between bacteria. Among the primary ways bacteria develop resistance are active drug efflux, decreased drug permeability, enzymatic drug inactivation, and modification of drug targets (33).

One of the most well-documented enzymatic mechanisms of resistance is through β-lactamases, which are enzymes that hydrolyze the β-lactam ring found in penicillin and related antibiotics. These enzymes render the antibiotics ineffective and are widely present in the ESKAPE group of pathogens, which include *Enterococcus faecium, S. aureus, Klebsiella pneumoniae, Acinetobacter baumannii, Pseudomonas aeruginosa,* and *E. coli* (34). These pathogens are particularly concerning due to their clinical importance and high resistance rates. Historically, resistance began to emerge shortly after the introduction of penicillin, leading to the appearance of β-lactamases like TEM-1, SHV-1, and TEM-2. While structurally similar, these enzymes differ in prevalence and effectiveness, but are generally inhibited by β-lactamase inhibitors such as clavulanic acid, sulbactam, and tazobactam (35).

The development of extended-spectrum β-lactamases (ESBLs) marked another phase of resistance evolution. These enzymes, which arose from point mutations in earlier β-lactamases, can hydrolyze a wide range of cephalosporins, posing a significant challenge to treatment (36). Additionally, resistance mechanisms such as enzymatic hydrolysis are also found against other classes of antibiotics like macrolides, rifampicin, and fosfomycin. Enzymes like esterases, ADP-ribosyltransferases, and chloramphenicol acetyltransferase modify or degrade these drugs, preventing their binding to ribosomal targets and thereby neutralizing their effects (37).

Furthermore, bacteria can develop resistance to antibiotics by modifying the target sites of drugs. A well-known example is the methylation of 23S rRNA in the 50S ribosomal subunit, which leads to resistance against macrolides, lincosamides, and streptogramin B (38). Changes in ribosomal proteins L4 and L22 or mutations in 16S rRNA also confer resistance to macrolides and aminoglycosides, respectively. These alterations reduce the ability of the active antimicrobial molecule to bind effectively its target without compromising the bacterial function, allowing the pathogen to survive antibiotic treatment (39).

Efflux pumps represent another major resistance strategy. These are membrane proteins that actively expel antibiotics out of bacterial cells, thereby reducing the intracellular concentration of the drugs. Efflux pumps are classified based on their energy source: primary active transport, which uses ATP hydrolysis, and secondary active transport, which relies on ion gradients (40). The ATP-binding cassette (ABC) transporters are well-studied examples of primary transporters, while families like the major facilitator superfamily (MFS), multidrug and toxic compound extrusion (MATE), and resistance-nodulation-cell division (RND) superfamily represent secondary active systems (41). These efflux mechanisms are not only essential for bacterial survival but also contribute to MDR in clinically important pathogens such as methicillin-resistant *S. aureus* and *Mycobacterium tuberculosis* (42).

The growing concern over AMR has been increasingly tied to the role of farm animals as significant reservoirs of ARB and ARGs. Numerous studies have documented that close contact with livestock, such as pigs, poultry, and cattle, can facilitate the transmission of resistant bacterial strains like *Salmonella typhimurium, E. coli, K. pneumoniae*, and *S. aureus* to humans (43). This transfer can occur directly, through physical interaction with animals or humans carrying resistant strains, or indirectly, through contaminated food products, polluted agricultural environments, and water sources associated with farm operations. Farm workers and veterinarians are particularly vulnerable due to their frequent contact with animals. A growing number of studies have shown that farm workers have higher rates of colonization with ARB than the general population (44).

Molecular studies revealed genetic similarities between resistant strains in humans and animals, supporting the hypothesis of cross-species transmission. For instance, strains of *K. pneumoniae* associated with urinary tract infections in humans have been genetically linked to bacteria found in retail meat, while a methicillin-resistant lineage of *S. aureus* appears to have originated in humans, acquired resistance traits in animals, and then re-entered the human population (45, 46). Experiments have confirmed such transmission pathways, including a foundational study where *E. coli* carrying MDR plasmids were spread among chickens and eventually appeared in human samples from farm personnel (47). These findings underscore the risks of antibiotic use in animal husbandry, especially as growth promoters, which creates selective pressure for resistant phenotypes (48).

Epidemiological research further demonstrates the zoonotic dimension of AMR. For instance, *mcr-1* genes, which confer resistance to colistin (a last-resort antibiotic), were found in both livestock and farmers, showing direct zoonotic transmission of high-risk ARGs (49, 50). Also, farmworkers and their household members were found to harbor high rates of ESBL-producing *E. coli* and *K. pneumoniae*, with shared resistance genes, indicating that close human-animal interaction plays a critical role in the transmission of resistant organisms (51).

Beyond direct contact, the high density of livestock in intensive farming systems facilitates the propagation of ARB. Antibiotics are excreted largely unmetabolized into the environment, contaminating soil and water, and sustaining ARGs within microbial communities in these ecosystems (52). This environmental contamination acts as a reservoir for resistance that can be transferred horizontally between bacterial species (53). This complex ecology of resistance is exacerbated by long-term and widespread antibiotic use in livestock activities, which significantly influences the diversity and abundance of ARGs within animal intestinal microbiomes (54).

### 3.3 The role of gut microbiome and metagenomics in tackling AMR

The implications of ARGs spreading are dreadful. According to the European Commission, AMR costs the EU alone over €1.5 billion annually in healthcare expenses and productivity losses. If left unaddressed, we may face a “post-antibiotic era” where common infections once again become untreatable (55). Veterinary medicine is also at risk, as the rise in ARB limits treatment options for animal diseases and endangers animal welfare (17). Alarmingly, some of the most potent last-resort antibiotics, like colistin and tigecycline, have been extensively used in animal agriculture, accelerating the development of AMR (56).

To fight this crisis, researchers are increasingly turning to metagenomic approaches, particularly the analysis of gut microbiomes in both humans and animals. These studies provide a comprehensive view of microbial communities, allowing scientists to trace the origins and pathways of AMR transmission (57). Such insights are crucial for designing targeted interventions and monitoring the effectiveness of antibiotic stewardship programs. Indeed, metagenomic studies enable researchers to comprehensively examine the gut resistome, which is defined as the collection of all ARGs within a microbial community (58). This technology allows to identify and characterize ARGs without the need for culturing individual microbes (59).

As widely described elsewhere, diet strongly shapes both the gut microbiome and resistome, influencing microbial composition and the selective pressures that drive antimicrobial resistance (60). For instance, animal-rich diets can shift microbial communities, increase secondary bile acids linked to inflammatory and metabolic disorders, and generate distinct responses depending on fat type (61–63). While nutrients such as tryptophan are converted into immunomodulatory metabolites like indole (64). Fermented dairy products may also deliver beneficial microbes and prebiotics that support *Bifidobacterium* and *Lactobacillus*, highlighting the diverse microbiota-mediated effects of animal-derived foods (65). Beyond nutrition, intestinal microbiomes of livestock and humans are interconnected across biological, ecological, as well as occupational dimensions. Phylogenetic studies reveal substantial microbial overlap between livestock and humans, particularly among individuals occupationally exposed to farm animals (66). Pivotally, workers in farming production exhibit higher fecal abundance of ARGs, reflecting horizontal gene transfer across species boundaries (67). This microbial exchange also has zoonotic implications: for instance, *Plasmodium* infections, while vector-borne, are known to reshape host gut microbiota, altering disease susceptibility and immune outcomes (68).

**Figure 2:**
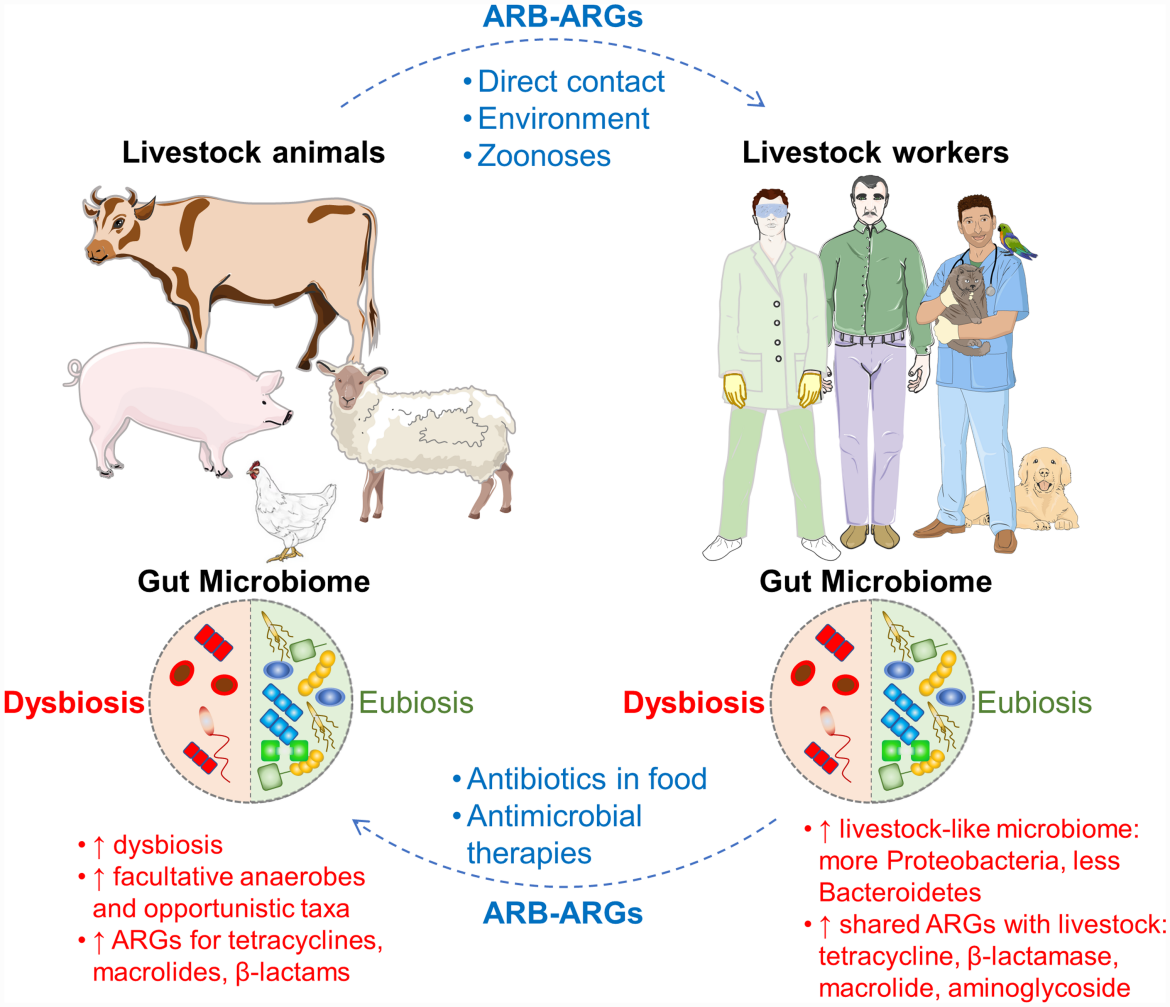
Cyclical transmission of antimicrobial-resistant bacteria (ARB) and antimicrobial resistance genes (ARGs) between livestock animals and occupationally exposed workers within a shared environment. The transmission of ARB and ARGs from livestock to workers may happen via direct contact, environmental exposure (e.g., contaminated surfaces, manure, and airborne particulates), and zoonotic pathways. Workers may contribute to the antimicrobial resistance (AMR) dissemination back to livestock through the use of antibiotics in feed, water, or veterinary antimicrobial therapies.

Within the gastrointestinal tracts, both livestock and workers might experience intestinal microbiome alterations. In livestock, the presence of ARB and ARGs can drive dysbiosis, which is characterized not only by increased levels of facultative anaerobes and opportunistic taxa, but also by elevated ARGs able to confer resistance to tetracyclines, macrolides, and β-lactams. In contrast, livestock workers often exhibit a livestock-like microbiome profile, marked by a rise in Proteobacteria, a reduction in Bacteroidetes, and the presence of ARGs shared with animals, including those encoding resistance to tetracyclines, β-lactamases, macrolides, and aminoglycosides (69). A summary of this intricate interrelation is shown in Figure 2.

For all these reasons the role of livestock gut microbiome as a reservoir for ARGs has emerged as a major public health concern. Metagenomic analyses have shown that ARGs can be transferred between commensal and pathogenic bacteria through horizontal gene transfer mechanisms such as conjugation, transformation, transduction, and vesiduction (70). This capability for gene exchange allows resistance traits to spread rapidly, even across taxonomically unrelated species, bypassing traditional barriers to gene flow (71).

The utility of metagenomic approaches lies in their ability to detect ARGs directly from complex microbial communities, offering insights into how antibiotic use selects for and amplifies AMR in the gut microbiome (72). Several studies demonstrated that antibiotic exposure significantly alters gut microbial composition, often reducing diversity and enabling ARB to dominate (73). Furthermore, these shifts facilitate the accumulation and persistence of ARGs, which can be transmitted through direct contact, environmental exposure, or consumption of contaminated food products (74).

Importantly, metagenomic studies not only uncover the mechanisms of AMR but also reveal the dynamics of gut microbial community composition in response to either acute or chronic antibiotic exposure. For example, antibiotic-induced dysbiosis often results in a reduction in beneficial short-chain fatty acid (SCFA)-producing bacteria, an increase in gut pH, and a competitive advantage for Gram-negative MDR bacteria (75). This disruption enhances the likelihood of resistant pathogen colonization and persistence, a trend observable across human populations, as well as animal models (76).

Metagenomic investigations have also evidenced the role of environmental and lifestyle factors (including diet, age, disease, and geographic location) in shaping the gut resistome (77). For instance, animals exposed to antibiotics in feed or water show increased ARG prevalence, which metagenomic tools can accurately quantify. Studies have found that animals fed with antibiotics like amoxicillin, thiamphenicol, or chlortetracycline exhibit significantly enriched ARG profiles in their gut microbiota (69). Heavy metals like copper and zinc, used as feed additives, also co-select for ARGs through mechanisms of co-resistance or cross-resistance, contributing to the complexity of gut resistome evolution (78).

One of the key strengths of metagenomic methods is their application within the One Health framework, which emphasizes the interconnectedness of human, animal, and environmental health. Metagenomics allows to trace ARGs across these domains, revealing shared resistance genes among microbiomes from different hosts and ecosystems, from humans to animals to environment (79). This insight is crucial for understanding how anthropic activities might drive the selection and spread of AMR across biological and geographical boundaries.

Finally, metagenomic studies have linked gut resistomes to various human health conditions beyond infections and zoonoses (80). Elevated ARG abundance has been associated with chronic diseases such as diabetes, cirrhosis, kidney disease, and even neurological disorders like autism spectrum disorder (81). These findings suggest that ARGs not only reflect antibiotic exposure, but it may also influence overall host physio-pathology in profound ways (82).

### 3.4 Gut microbiome analyses reveal mechanisms AMR spread in livestock

Recent integrative research into the gut microbiomes and resistomes of livestock systems revealed a complex host- and environment-dependent landscape of AMR, shaped by microbial ecology, host genetics, antibiotic use, and horizontal gene transfer (Table 3).

**Table 3:** Summary of livestock gut microbiome and resistome findings across animal studies.

Animal (type, number)	Specimen (type, number)	Microbiome outcomes	Resistome outcomes	DOI
Ruminants (dairy and beef cattle, sheep; number not specified)	Rumen contents (435 genome assemblies)	Dominant bacterial phyla: Firmicutes, Proteobacteria, Bacteroidetes, Actinobacteria; Enterobacteriaceae exhibited the highest ARG density	High prevalence of tetracycline resistance genes; active ARG expression confirmed; MGEs implicated	(83)
Dairy calves (*n* = 22)	Feces (*n* = 484), maternal colostrum	Early dominance of Enterobacteriaceae and anaerobic taxa (Ruminococcaceae, Lachnospiraceae, Bacteroidaceae) reflecting dietary progression	329 unique ARGs spanning 17 antibiotic classes; age-related decline in overall ARG abundance; persistent tetracycline and macrolide ARGs; vertical ARG transmission via colostrum	(84)
Cattle (number not specified)	Feces, soil, and wastewater (*n* = 128)	Fecal microbiota dominated by Bacteroidetes and Firmicutes; soil by Actinobacteria and Proteobacteria; wastewater by Proteobacteria	Detected 440 ARGs across 16 antibiotic classes; higher ARG abundance in conventional systems; tetracycline and macrolide genes dominant in feces; β-lactam genes enriched in dairy cattle; resistome diversity weakly linked to microbiome composition	(85)
Piglets (*n* = 19 for transcriptomics; *n* = 47 for metagenomics)	Ileal and colonic contents (*n* = 47)	Overall microbial diversity stable; increased abundance of *B. fragilis* observed post-antibiotic exposure	Identified 1,021 ARGs across 39 antibiotic classes; moderate enrichment of ARGs corresponding to administered antibiotics (chlortetracycline, virginiamycin); modest increase in MGEs; targeted selection without global resistome restructuring	(86)
Pigs (*n* = 467, pooled from 38 farms)	Feces, soil, and slurry (*n* = 105)	Stronger microbiome-resistome correlation in high-antibiotic-use systems; industrial systems showed distinct microbial profiles	Greater resistome abundance and diversity in environmental matrices (slurry, soil) vs. feces; tetracycline ARGs highly diverse; β-lactam ARGs clonally expanded; plasmids and integrons enriched in intensive systems	(87)
Holstein dairy cows (*n* = 416)	Rumen contents (across 14 farms)	Taxonomic structure spanning 15 bacterial phyla; core microbiome associated with macrolide and tetracycline resistance	Detected 998 distinct ARGs; major mechanisms included efflux pumps and tetracycline resistance proteins; strong phage-ARG associations; resistome composition partially heritable via host genetics	(88)
Dairy ruminants (*n* = 31: 16 cows, 15 buffaloes)	Rumen contents (*n* = 31)	Buffaloes exhibited higher bacterial richness; enriched in *P. ruminicola* and *L. amylovorus*; increased carbohydrate-active enzyme gene content	505 ARGs identified; buffaloes exhibited greater ARG diversity and abundance; predominant resistance mechanisms included efflux, enzymatic inactivation, and target replacement	(89)
Pigs (*n* = 30), Poultry (*n* = 60)	Feces (samples from Ghana and 9 European countries)	Microbiome structure shaped by host species and geography; Ghanaian poultry enriched in *Subdoligranulum*, *Streptococcus*, *Olsenella*	Ghanaian poultry had highest ARG abundance (dominated by tetracycline genes); European livestock had higher macrolide, β-lactam, and trimethoprim resistance; aminoglycoside resistance enriched in Ghanaian pigs	(90)

For instance, genome-resolved metagenomic and metatranscriptomic analyses by Vieira Sabino et al. demonstrated the dominance of Firmicutes, Proteobacteria, Bacteroidetes, and Actinobacteria in ruminant microbiomes. Also, the authors found that tetracycline resistance genes, particularly tetW, were selected and maintained within the population, and were carried by novel integrative conjugative elements, capable of transferring resistance genes between bacteria (83). Functional assays and transcriptomics confirmed active expression of resistance genes, implicating the rumen as a functionally active resistome reservoir. Among all gastro-intestinal microorganisms, Enterobacteriaceae exhibited the highest ARG density, with *Bacteroides* and *Enterococcus* contributing to taxonomic resistome heterogeneity (83).

Longitudinal profiling by Liu et al. in neonatal dairy calves revealed rapid gut microbial shaping and dynamic resistome restructuring during early life, with a shift from *E. coli*-dominated communities to obligate anaerobes such as Ruminococcaceae and Lachnospiraceae as the diet transitioned from colostrum to solid feed (84). Although overall ARG abundance declined with age, certain ARGs, especially against tetracyclines and macrolides, persisted or even increased. Maternal colostrum was identified as a reservoir for early-life ARGs, including *E. coli* strains carrying them, supporting the vertical transmission hypothesis. Co-selection via co-occurring metal and biocide resistance genes was also observed, reinforcing the role of non-antibiotic selective pressures within resistome maintenance mechanism (84).

Besides, Rovira et al. characterized resistome variation across conventional and antibiotic-free cattle production systems analyzing 128 samples from feces, soil, and wastewater. Microbiome composition varied significantly by matrix type, while resistome profiles were more closely linked to antibiotic usage. Environmental samples from conventional systems showed higher ARG richness and abundance, especially for tetracyclines and macrolides, whereas β-lactam resistance genes predominated in dairy cattle gut resistome (85). Soil resistomes exhibited unique ARG profiles and were less affected by antibiotic use, suggesting that environmental compartments may act as independent ARG sources. Moreover, weak correlations between microbial community structure and resistome composition emphasize the central role of selective pressure over community composition in shaping ARG diversity (85).

Through the use of a controlled piglet model, Hu et al. evaluated the effects of low-dose chlortetracycline and virginiamycin exposure. While global gut microbial diversity remained stable, transcriptomics indicated a reduction in gut inflammation and enhanced epithelial barrier function (86). Despite no significant change in overall resistome diversity or abundance, specific ARGs (particularly those targeting widely-administered antibiotics) were found enriched, and MGEs slightly increased. These findings illustrate that even low-dose antibiotics can selectively amplify particular ARGs without major shifts in the resistome structure (86).

Additionally, Mencía-Ares et al. compared pigs raised in intensive industrial systems versus extensive Iberian farming, identifying higher resistome abundance and diversity in high-antibiotic-use environments, particularly within environmental matrices, such as slurry and soil (87). Tetracycline ARG diversity and β-lactam ARG abundance were elevated in intensive systems, reflecting intensified selective pressures. Key ARG hosts were found in feces, including Streptococcaceae, Bacteroidaceae, and Enterobacteriaceae, with enriched plasmids and integrons supporting increased horizontal gene transfer. Strong associations between antimicrobial use and ARG prevalence further underscore the role of management practices in shaping resistome complexity (87).

Also, López-Catalina et al. conducted a high-resolution resistome survey in Holstein dairy cows, revealing a total of 998 ARGs across 15 microbial phyla. The core resistome was dominated by macrolide and tetracycline resistance genes, with efflux pump mechanisms accounting for a significant portion of ARG abundance (88). Extensive ARG co-occurrence networks and associations with bacteriophages highlighted phage-mediated horizontal gene transfer as a major dissemination route. Host genetic influences on resistome composition were also detected, and the persistence of ARGs against discontinued antibiotics indicated co-selection independent of current antimicrobial utilization (88).

In a comparative study analyzing dairy cows and buffaloes, Sun et al. demonstrated species-specific resistome and microbiome profiles. In particular, buffaloes exhibited higher bacterial loads and greater ARG diversity, including several unique ARGs not observed in cows. For instance, the tcmA gene, associated with tetracenomycin resistance, was particularly enriched and co-expressed with *Lactobacillus* spp., suggesting a link between host physiology and ARG prevalence (89). Dominant resistance mechanisms included efflux, target modification, and enzymatic inactivation, emphasizing the role of host species in structuring resistome architecture (89).

Furthermore, Jensen et al. extended the geographical scope of resistome analyses by comparing livestock from Ghana and nine European countries. Ghanaian poultry harbored the most diverse and abundant ARGs, especially tetracycline resistance genes, while Ghanaian pigs exhibited lower ARG levels than their European counterparts (90). Multivariate analyses confirmed host species as the primary determinant of resistome and microbiome composition, with farming practices exerting a more pronounced effect in poultry than in pigs. Overall, differences in ARG profiles across regions reflected both biogeographic and management influences on AMR ecology (90).

Collectively, these studies reveal that the both gut microbiome composition and abundance, as well as gut resistome in livestock are shaped by a confluence of factors including: host species, age, antibiotic exposure, production system, and environmental context. Also, horizontal gene transfer via MGEs (such as plasmids, integrons, and bacteriophages) emerges as a central mechanism in ARG dissemination. Pivotally, the persistence observed in certain studies of ARGs unrelated to current antibiotic use highlights the importance of co-selection and ecological maintenance. This body of evidence suggests the need for a systems-level approach to AMR surveillance and mitigation, integrating microbial ecology, host genetics, and environmental reservoirs within One Health frameworks, in order to better address ARG emergence and spread across livestock systems (91).

### 3.5 Occupational livestock exposure shapes human gut microbiome and resistome

Over the past decade, an increasing body of research explored how occupational exposure to livestock and farming environments affects the human gut microbiome and resistome, highlighting the complex interplay between microbial communities, ARGs, and human health (Table 4).

**Table 4:** Comparative studies on gut microbiomes and resistomes in livestock-exposed workers.

Human population (type, specimen, N)	Animal population (type, specimen, N)	Environmental samples (type, N)	Study type	Gut microbiome composition	Gut resistome composition	Exposure effect on human samples?	Country	DOI
Farm workers (feces, 6), local villagers (feces, 6)	Swine (feces, 6)	None	Cross-sectional	Workers gut dysbiosis, lower diversity than villagers; workers’ microbiota resembled swine (↑Proteobacteria, ↓Bacteroidetes)	Swine resistome matched antibiotics used; human resistome broader, limited ARG transfer	Occupational exposure shifts microbiome toward swine-like, limited ARG transfer	China	(92)
Vet students (feces, 14), farm workers (feces, 5), urban controls (feces, 196)	Swine (feces)	Dust, swine feces, sewage, soil	Longitudinal (9 months)	Shift in workers: ↓Bacteroidetes, ↑Proteobacteria, some recovery post-exposure	1924 ARGs detected; subtle increase in β-lactamases, tetracycline resistance; ARGs overlap with environment; clonal transmission	Exposure caused dynamic microbiome and resistome shifts, some ARG persistence	China	(93)
Women exposed (feces, 5), non-exposed (feces, 6)	Broiler chickens (feces)	None	Longitudinal (12 months)	Not detailed	Chickens had 23 AMR genes; humans shared ESBL genes; AMR pattern similarity higher in exposed women	Exposure associated with AMR pattern similarity; directionality unclear	Uganda	(94)
Farmers (feces, 33), controls (feces, 46)	Broiler and pig farms (feces, 96)	Indoor farm dust (96)	Cross-sectional	Farm dust microbiome distinct from feces/human; farmers gut microbiome stable	Dust had higher ARG diversity than feces/human stool; dust ARGs overlapped feces but farmers gut resistome stable	Dust is ARG reservoir but no major changes in farmers gut resistome	Europe (9 countries)	(95)
Workers (pig/broiler/slaughterhouse, feces, 194), controls (feces, 46)	Pig, broiler (feces)	None	Cross-sectional	Gut microbiomes varied with livestock exposure; pig slaughterhouse workers had reduced diversity	Pig-exposed workers had higher ARG loads (tetracycline, β-lactam, macrolide); resistome linked to exposure intensity	Occupational exposure elevated ARG carriage independently of antibiotic use	Netherlands	(96)
Farmers (feces), controls (feces)	Swine (feces)	None	Cross-sectional	Farmers’ gut microbiota intermediate between swine and controls; Firmicutes/Bacteroidetes dominant	Metabolome stable despite microbiome shifts; butyrate elevated in swine; no major metabolic change	Exposure partially converged microbiota but metabolome stable	Malaysia	(97)
Live poultry market workers (feces, 18), controls (feces, 18)	Chickens (feces, pooled 135 samples)	Environmental samples from farms and markets	Cross-sectional	Workers’ gut microbiota distinct from controls; chicken gut dominated by Firmicutes, Bacteroidetes, Proteobacteria	Workers had elevated ARG diversity (188 ARG types), including mobile colistin resistance genes	Occupational exposure reshapes microbial communities, elevates ARG diversity	China	(98)
High exposure (wet market, backyard poultry, feces, 8), low exposure (feces, 12)	Poultry (cecal samples, 10)	Wastewater (10)	Cross-sectional	Human gut microbiomes stable; dominated by anaerobic commensals (*Prevotella*)	Poultry/wastewater had high ARG diversity; wastewater major ARG reservoir; minimal direct ARG transfer to humans	Poultry exposure did not significantly affect human ARG carriage	Bangladesh	(99)
Humans (healthy + *C. difficile* infected, feces, 87)	Swine, cattle, chickens (feces, 108)	None	Cross-sectional	Host-specific microbiomes; patients had distinct taxa from healthy; livestock had own signatures	ARG abundance higher in patients; shared ARGs between poultry and humans indicate cross-species transmission	Poultry exposure linked to resistome reshaping and ARG dissemination	South Korea	(100)
Swine farming village residents (feces, 36), non-exposed villagers (feces, 18)	Swine (feces)	Soil, air, groundwater, river water	Cross-sectional	Increased diversity and dysbiosis markers in exposed residents; ↑Proteobacteria (*K. pneumoniae*, *E. coli*)	376 ARGs detected; 144 unique to exposed; ARGs linked to swine feces (31%) and airborne sources (20.5%)	Exposure significantly alters microbiome and expands resistome via environment	China	(101)
Dairy workers (feces, 10), non-farming controls (feces, 6), HMP reference (47)	None	None	Cross-sectional	Similar gut microbiome taxa across groups; dairy workers had lower functional richness	Dairy workers had higher tetracycline and cephamycin resistance genes; no overall ARG abundance difference	Subtle occupational impacts on resistome, especially specific ARG classes	United States	(102)
Dairy farmers (feces, 66), controls (feces, 60)	Dairy cows (feces, 166)	None	Longitudinal (13 months)	Farmers gut similar to controls but some livestock-associated taxa enriched	>2,000 novel ARGs from cows; farmers harbored distinct ARGs shared with livestock; seasonal ARG fluctuations	Occupational exposure influences resistome and functional microbiome capacity	United States	(103)
Various human groups (feces, total 504): vegans, students, workers, farmers, omnivores, abstainers	Poultry (feces, 24), Swine (feces, 20)	Soil (10), surface water (9), wastewater (4), flies (20), food (51)	Cross-sectional	Human gut dominated by Bacillota and Bacteroidota; environment enriched with Pseudomonadota	Food and environment had higher ARG burden; shared ARG lineages across sources; occupational and diet influence	Occupation, diet, habitat shape microbiome and resistome; environment and vectors critical ARG sources	China	(104)

These studies, spanning diverse geographic regions and animal production systems, collectively reveal heterogeneous impacts of farming on gut microbiome composition, ARG prevalence, and potential risks for both workers and work-surrounding communities. One of the earlier comprehensive investigations conducted in China, compared stool-derived microbiome from 6 swine treated with tetracycline and sulfonamide antibiotics, 6 farm workers, and 6 local villagers with no recent livestock contact (92). This study found that the gut microbiome composition differed between groups. Notably, farm workers exhibited signs of gut dysbiosis, including lower microbial diversity and richness compared with villagers, with their microbiota more closely resembling that of swine. Specifically, both swine and workers showed increased Proteobacteria and decreased Bacteroidetes relative to villagers, along with elevated Enterobacteriaceae, Clostridiaceae, and Lachnospiraceae (92). The resistome analysis revealed that swine ARG profiles matched administered antibiotics (e.g., tetracycline, lincomycin, sulfanilamide), while human resistomes were broader, but with limited direct ARG transfer between swine and workers. These findings suggested that occupational exposure shifts the human gut microbiome toward swine-like communities. However, the assumption of systematic ARG transmission through direct contact remains challenging to proof (92).

A longitudinal study of 14 veterinary students undergoing farm exposure and comparisons with 5 full-time farm workers and 196 healthy non-exposed urban controls in China further illustrated dynamic microbiome and resistome shifts (93). Within 1 month of farm exposure, students’ gut microbiota shifted significantly, with decreased Bacteroidetes and increased Proteobacteria (notably Gammaproteobacteria), although overall alpha diversity remained stable. Specific genera such as *Faecalibacterium*, *Collinsella*, and *Blautia* show variability in their abundance, with partial recovery observed post-exposure (93). Also, changes in the resistome were extensive, detecting a total of 1924 ARGs in the human gut, including β-lactamases, aminoglycoside resistance genes, and chloramphenicol acetyltransferases. Although overall ARG abundance remained stable within the different populations, subtle increases in β-lactamases and tetracycline resistance were noted in the students’ gut resistome during or after swine farm stays (93). Importantly, approximately 40% of new gut genes overlapped with the farm environment (dust, swine feces, sewage, soil), and clonal bacterial transmission was demonstrated, including the detection of ARB carrying ARGs alongside virulence and biocide resistance genes. Also, some ARGs persisted in student’s human guts for 6–9 months after exposure, highlighting occupational risks and the environmental role in ARG dissemination and persistence (93).

Similar patterns regarding resistome outcomes emerged from a Ugandan study investigating women exposed to small-scale poultry farms (94). The study involved 5 exposed women who were exposed to broiler and 6 non-exposed ones. Gut microbiome from chickens carried a total of 23 AMR genes, including those for β-lactams, vancomycin, and tetracyclines, with vancomycin and carbapenemase genes exclusive to chickens (94). Human microbiota harbored diverse AMR genes, including tetracycline resistance genes (tetA, tetB), and shared β-lactamase ESBL genes, suggesting common resistance mechanisms. Intriguingly, women who had direct contact with the chickens showed more similarities in AMR gene patterns to the chickens compared to those who did not have direct contact (94). However, after a year, there was a tendency for increased similarity in AMR patterns between humans in both groups and the chickens sampled. Due to the timing of sample collection, the directionality of AMR gene transmission between poultry and humans remained unclear, underscoring the complexity of environmental and community sources in their contribution to human resistome shaping (94).

In Europe, a large-scale study sampling several broiler and pig farms across nine countries demonstrated that airborne farm dust harbors highly diverse microbiomes distinct from animal fecal and human gut microbiomes, with bacterial taxa reflective of the farm animal species (95). Samples collected included stool from farmers (*n* = 33) and non-exposed controls (*n* = 46), as well as 96 animal feces and indoor farm dust. The bacterial communities in farm dust differed significantly from those in animal feces and human gut samples, containing taxa such as bacilli and clostridia in poultry farms and Betaproteobacteria in pig farms, indicating a composite environmental origin including skin, feed, soil, and other animals beyond fecal sources. Despite occupational dust exposure, the gut microbiome of farmers remained largely stable and distinct from environmental samples, suggesting limited impact on the human gut bacterial community (95). Regarding gut resistome composition, farm dust was identified as a rich and diverse reservoir of ARGs, exceeding the diversity found in both animal feces and human stool samples. Many ARGs in dust overlapped with those in feces from the corresponding animal species, underscoring fecal shedding as a major contributor to the airborne resistome (95). However, dust also contained unique ARGs not detected in feces or human samples, including chloramphenicol-florfenicol and β-lactamase ARGs, indicating additional environmental sources. The resistome composition in dust correlated strongly with bacterial community structure and it was positively associated with farm antimicrobial use, though this relationship was mainly mediated through fecal ARG abundance (95). Despite the presence of this environmental resistome reservoir, the gut resistome of farmers showed no significant shifts related to dust exposure, clustering distinctly from environmental samples and sharing fewer ARGs with dust or animal feces (95).

A related study in the Netherlands involving 194 workers (including pig farmers, broiler farmers, slaughterhouse workers) and 46 non-exposed controls, revealed that gut microbiomes varied significantly with livestock exposure (96). In terms of gut microbiome composition, dominant bacterial phyla such as Bacteroidetes, Firmicutes, and Actinobacteria were consistently present across all groups. However, the relative abundance and diversity of specific genera varied significantly depending on livestock exposure. Notably, pig slaughterhouse workers exhibited reduced alpha diversity of their gut bacteriome compared to other groups, with shifts in key genera such as *Prevotella* and *Bacteroides*, indicating altered microbial richness (96). Regarding gut resistome, pig farmers and slaughterhouse workers showed significantly higher total ARG loads compared to broiler farmers and controls. This increase was especially evident for ARGs conferring resistance to tetracyclines, β-lactams, and macrolides, which dominated the resistome landscape. Although alpha diversity of ARGs was similar across groups, beta diversity analyses highlighted distinct ARG profiles associated with both pig and pork exposure. A total of 30 ARGs were significantly enriched in pig-exposed workers relative to controls, with many belonging to the dominant resistance classes, reflecting a clear occupational signature. Importantly, resistome differences persisted after controlling for antimicrobial use and they were linked to factors such as job role and duration of animal contact, emphasizing exposure intensity as a critical driver. Hence, this study underscored that occupational exposure elevates ARG carriage independently of recent antibiotic use (96).

Research from Malaysia showed that swine-exposed farmers had gut microbiota profiles intermediate between swine and unexposed controls, with alpha diversity highest in swine and lowest in controls (97). Bacteroidetes, Firmicutes, and Proteobacteria dominated across groups, although the Firmicutes-to-Bacteroidetes ratio was highest in swine, and *Prevotella* (particularly *Prevotella copri*) was the most abundant genus. Despite these taxonomic shifts, the metabolome remained stable, indicating functional resilience of the gut ecosystem. Butyrate levels were elevated in swine but showed no clear taxonomic association, suggesting a collective microbial contribution. Overall, the study demonstrated that occupational swine contact led to partial convergence of the gut microbiota of farmers toward swine-like profiles without major functional changes (97).

A study focused on live poultry markets in China analyzed fecal samples from 18 workers, 18 non-exposed human controls, alongside with 135 pooled chicken fecal samples (representing over 1,200 chickens), and environmental samples from farms and markets (98). In terms of gut microbiome composition, chicken gut microbiota was predominantly composed of Firmicutes, Bacteroidetes, Proteobacteria, and Actinobacteria. Microbial richness strongly correlated with ARG diversity, highlighting the role of the microbiome in shaping resistome profiles (98). Notably, microbial and resistome diversity were significantly higher in live markets compared to commercial farms. Geographic variation was also observed, suggesting local farming and trade practices influence microbial and resistome dynamics. Importantly, live market workers displayed distinct gut microbiota compositions relative to controls living in the same region, with occupational exposure implicated in reshaping intestinal microbial communities. Also, workers exhibited elevated ARG diversity with 188 unique ARG types, including mobile colistin resistance genes present in both chickens and humans. The frequent co-occurrence of ARGs and MGEs highlighted live poultry markets as critical hotspots for zoonotic ARG transmission, including resistance to last-resort antibiotics like colistin (98).

A more recent study in Bangladesh conducted a detailed metagenomic analysis of fecal samples from 8 high exposed subjects (i.e., wet market workers and backyard poultry owners) and 12 low exposed subjects (i.e., food vendors, and non-poultry rural households), 10 poultry cecal samples and 10 wastewater samples (99). The research revealed that while human gut microbiomes remained stable and dominated by anaerobic commensals (i.e., *Prevotella*), poultry exposure did not significantly affect human ARG carriage. Poultry and wastewater samples harbored high ARG diversity, with wastewater identified as a major environmental reservoir and amplifier of clinically relevant ARGs such as ESBLs and carbapenemases (99). This indicates minimal direct ARG transfer from poultry to humans in this context but highlights the critical role of wastewater in ARG dissemination. Indeed, the human fecal resistome did not significantly differ between individuals with high versus low occupational poultry exposure, indicating that direct ARG transmission from poultry to humans under the studied conditions was limited (99).

In a comparative Korean study, assessing fecal samples from 87 humans (healthy and *Clostridioides difficile* infection patients) and 108 livestock (swine, cattle, chickens), distinct microbial communities were found to be host-specific (100). In details, healthy individuals were characterized by predominance of *Bifidobacterium*, *Ruminococcus*, and *Bacteroides*. Patients with *C. difficile* infection showed higher levels of *Enterococcus*, *Lactobacillus*, and *Bacteroides*. Interestingly, swine guts were dominated by *Prevotella*, *Lactobacillus*, and *Subdoligranulum*, whereas cattle harbored mainly *Peptostreptococcus*, *Butyrivibrio*, and *Treponema*, and, finally, chicken gut microbiota was enriched in *Bacteroides*, *Alistipes*, and *Barnesiella* (100). Regarding the resistome, ARG abundance was more than four times higher in *C. difficile* patients than in healthy individuals, and chickens showed ARG diversity comparable to infected patients. Shared ARGs such as β-lactamase and quinolone resistance genes suggested cross-species transmission, implicating occupational or environmental poultry exposure as vectors facilitating ARG dissemination to humans with consistent gut resistome reshaping (100).

A Chinese study demonstrated that human residents exposed to swine farming had significant changes in microbiome and resistome. In details, the analysis encompassed human gut samples from 36 swine farming village residents and 18 non-exposed subjects form a non-farming village, alongside environmental specimens from soil, air, groundwater, river water, and swine feces, integrating microbial community profiling with resistome characterization (101). Compared to non-exposed subjects, the swine farmers microbiomes showed increased microbial diversity alongside markers of dysbiosis, including a reduced Gut Microbiome Health Index (GMHI) and an elevated presence of potentially pathogenic taxa (101). Notably, there was a significant enrichment of Proteobacteria (particularly *K. pneumoniae* and *E. coli*), as well as increased abundances of bacterial families such as Enterobacteriaceae, Rikenellaceae, and Eggerthellaceae. These shifts suggest that proximity to swine farming and associated occupational or environmental exposure contribute to perturbations in gut microbial ecology (101). The gut resistome analysis revealed a total of 376 ARGs were detected, with 144 unique to the farming village residents (i.e., enriched resistance to β-lactams, macrolides, fluoroquinolones, tetracyclines, and cephalosporins) often co-occurred with MGEs such as plasmids and integrons, which facilitate the horizontal transfer of resistance (101). Resistome attribution analysis indicated that approximately 31% of the resistome was linked to swine feces and 20.5% to airborne sources contributions, underscoring the significant role of environmental and airborne ARG transmission in elevating ARG exposure and potential health risks in farming communities. Overall, this study reveals that swine farming significantly alters the human gut microbiome and expands the gut resistome through environmental exposure, particularly via air and animal waste (101).

In dairy farming contexts, a further US study compared gut microbiome from stool samples from 10 dairy workers and 6 non-farming controls, with 47 additional samples from the Human Microbiome Project (HMP) serving as a reference cohort (102). In terms of gut microbiome composition, both dairy workers and controls exhibited similar taxonomic profiles dominated by the phyla Firmicutes, Bacteroidetes, and Actinobacteria, which are commonly associated with a healthy human gut. However, gene-level analyses revealed that dairy workers harbored lower functional genomic richness, indicating a potential occupational impact on the metabolic or functional potential of the gut microbiota, despite taxonomic similarity (102). While no overall ARG abundance differences were observed, dairy workers showed relatively higher tetracycline and cephamycin resistance genes, found in both commensal and potential pathogenic bacteria, suggesting subtle occupational impacts potentially linked to antibiotic use in livestock. Importantly, the presence of ARGs in commensal bacteria further highlights the gut as a potential reservoir for AMR (102).

A longitudinal 13 months-long American study systematically investigated the effects of occupational exposure to livestock on gut microbiome samples from 66 dairy farmers, 60 matched controls, and 166 cows across 37 dairy farms (103). The study revealed that dairy farmers’ nasal microbiomes were more diverse and closely resembled those of cows, while gut microbiome differences were subtle but included acquisition of livestock-associated species and functional pathways. Intriguingly, cows exhibited substantially higher gut microbial diversity overall, but farmers’ gut microbiomes showed enrichment in health-associated Firmicutes such as *Coprococcus eutactus* and *Roseburia faecis*, which are known butyrate producers (103). Although broad taxonomic structure remained similar between farmers and non-farmers, lineage-resolved analyses revealed that certain microbial taxa, including *Prevotella*, *Treponema*, *Romboutsia*, and *Bifidobacterium*, were significantly enriched in both cows and farmers. These taxa were also observed in the nasal microbiomes of both species, indicating interspecies microbial sharing potentially facilitated by close environmental contact (103). The resistome analysis identified over 2,000 novel ARGs predominantly from cows, with farmers harboring distinct ARGs shared with livestock, especially those conferring resistance to lincosamides, macrolides, phenicols, and tetracyclines. Moreover, seasonal increases in certain ARGs among farmers suggested ongoing cross-species ARG transfer and occupational exposure profoundly shaping both nasal and gut microbiomes. Collectively, the findings indicate that occupational exposure to livestock, while having limited impact on broad taxonomic diversity in the gut, significantly influences functional capacities and resistome content at a higher resolution (103).

To investigate gut microbial and resistome patterns, transmission vectors, and reservoirs, a large Chinese study analyzed 592 samples from human and animal feces, food, soil, water, and flies (104). Human fecal samples included individuals with different occupations and diets: vegan communes (*n* = 68), boarding students (*n* = 48), food processing workers (*n* = 50), livestock farmers (*n* = 50), omnivores (*n* = 49), vegetarians (*n* = 49), pork abstainers (*n* = 50), chicken abstainers (*n* = 46), and aquatic product abstainers (*n* = 44). Environmental samples (*n* = 87) included soil (*n* = 10), surface water (*n* = 9), wastewater (*n* = 4), flies (*n* = 20), poultry feces (*n* = 24), and swine feces (*n* = 20). Food samples (*n* = 51) comprised pork (*n* = 13), chicken (*n* = 11), and vegetables/fruits (*n* = 27) (104). For what concerns gut microbiome composition, human gut microbiomes were dominated by Bacillota (Firmicutes) and Bacteroidota (Bacteroidetes), while food and environmental samples were enriched with Pseudomonadota (Proteobacteria). Notably, food and environmental samples carried a higher ARG burden than human guts. *E. coli* played a central role in ARG dissemination, with extensive plasmid- and phage-mediated ARG mobility observed. Carbapenemase genes, conferring resistance to critical antibiotics, were widespread in all samples. Livestock farmers, in particular, harbored a higher load of phenicol resistance genes, suggesting occupational exposure as a critical factor in shaping the gut resistome (104). In contrast, individuals abstaining from pork exhibited lower levels of glycopeptide resistance genes, indicating dietary influence. Strain-level analyses revealed shared lineages of *E. coli*, *K. pneumoniae*, and *Clostridium perfringens* between humans and other sources, further substantiating the potential for cross-host and environmental transmission of antibiotic-resistant bacteria (104). The study highlighted the human gut as a key ARG reservoir and transmission hub within a broader ecological network, with flies acting as vectors, reflecting complex environmental and dietary exposures driving ARG transmission. It further corroborated the role of occupation, but also diet, and habitat in shaping both the gut microbiome and resistome, with important implications for AMR surveillance and intervention strategies (104).

Collectively, these studies illustrate a complex landscape where occupational exposure to livestock and farming environments can alter human gut microbiomes, increase ARG diversity, and facilitate microbial and ARG transmission. However, direct ARG transfer is often subtle and context-dependent, influenced by environmental reservoirs such as dust, wastewater, and air, as well as factors like animal species, farming practices, and regional antibiotic use (105). The findings emphasize the importance of integrated One Health surveillance encompassing humans, animals, and environmental sources to better understand and mitigate antibiotic resistance risks associated with agricultural exposures (7).

## 4 Conclusion

AMR remains a critical global health challenge, with livestock production systems acting as major reservoirs and transmission hubs for resistant microorganisms. Accumulating evidence robustly supports the central role of the gut microbiome in the emergence, persistence, and dissemination of ARGs both within animal populations and across species barriers. Moreover, current advances in metagenomic technologies have significantly deepened our understanding of these complex microbial ecosystems, elucidating the mechanisms driving AMR spread and underscoring the public health risks posed by occupational exposure to livestock environments (106).

This review has addressed pivotal questions at the intersection of gut microbiome dynamics, AMR development, and their broader implications for livestock farming and occupational health. Firstly, the gut warrants focused attention due to its well-established function as a hotspot for microbial communities and AMR genes. Depending on host health and environmental factors, gut microbial composition may facilitate the proliferation of ARB and promote horizontal gene transfer of ARGs among diverse species, thereby serving as a key reservoir for the emergence, amplification, and transmission of AMR within and between hosts.

Secondly, the current metagenomic data presented here highlighted a tightly interconnected ecological relationship between gut microbial communities and resistome profiles across both animal and human hosts. Dominated by phyla such as Firmicutes, Bacteroidetes, and Proteobacteria, the gut microbiota not only shapes the composition of the intestinal resistome but is itself modulated by it, reflecting bidirectional interactions. These dynamics reveal how antimicrobial usage, host species differences, and environmental contexts collectively influence AMR patterns. Notably, while ARG profiles are frequently host-specific, emerging evidence points to partial resistome overlap between humans and animals, especially in high-exposure occupational settings, indicating plausible routes for cross-species ARG transmission.

Despite these advances, methodological challenges within metagenomics techniques remain. Sequencing platform choice, coverage depth, and bioinformatic pipelines affect ARG detection and quantification (107). Reference databases may be incomplete or biased, and short-read sequencing can limit resolution of MGEs or accurate ARG-host assignment (108). Functional metagenomics and long-read sequencing can mitigate some limitations but involve trade-offs in cost and throughput (109, 110). Recognizing these constraints is crucial for accurate interpretation and effective AMR surveillance.

To reduce AMR consolidation, mitigation strategies aligned with One Health show promise (111–113). Alternatives to antimicrobial growth promoters, including phytogenic feed additives, selected probiotics, prebiotics, and enhanced husbandry practices, can modulate gut microbiota and reduce resistome burden (114–120). Environmental interventions, such as manure composting, further lower ARG abundance and limit dissemination (121–124). Indeed, effective AMR control requires integrated approaches combining antimicrobial stewardship, microbiome-based interventions, and improved environmental management (91).

On these bases, the One Health paradigm is quickly evolving toward integration with Planetary Health, emphasizing the interdependence of human, animal, and ecosystem health. Modern One Health recognizes that sustainable health outcomes depend on coordinated action across sectors and disciplines, addressing not only AMR and zoonoses but also climate change, food and water security, and ecosystem integrity (125).

In conclusion, addressing AMR requires integrated One Health-Planetary Health frameworks that monitor microbiomes, resistomes, and exposure pathways across humans, animals, and the environment (72). Targeted occupational interventions, improved sanitation, personal protective equipment, longitudinal monitoring of gut resistomes, and strengthened biosecurity are critical measures. Beyond veterinary or public health considerations, AMR management is a planetary imperative, demanding sustainable, collaborative action to safeguard the health of people, animals, and ecosystems (126).
